# Supplementary material for: Selection of Enzymatic Treatments for Upcycling Lentil Hulls into Ingredients Rich in Oligosaccharides and Free Phenolics
Source: Molecules. 2022 Dec 2;27(23):8458. doi: 10.3390/molecules27238458 (PMC9738424; doi:10.3390/molecules27238458)
Supplement: Supplementary file 1 [file molecules-27-08458-s001.zip › molecules-2045767-supplementary.pdf]

Table S1. Origin, working pH and temperature range and declared enzymatic activities of seven commercial glycosidases (Novozymes, Bagsværd, Denmark)

Enzyme	Origin	Working pH	Working Temperature (°C)	Declared activities
Ultraflo XL	*Humicola insolens*	4.0–6.5	45–70/75	Endo beta-glucanase (45 BGU /g). Xylanase, cellulase, pentosanase, arabinofuranosidase are declared as side activities
Ultraflo Max	*Aspergillus oryzae Trichoderma reesei*	4.0–6.5	45–70/75	β-glucanase (endo-1,3(4)-) (700 EGU/g) and Xylanase (endo-1,4-) (250 FXU/g)
Ultimase BWL 40				Cellullase (1200 CIU/g)
Viscozyme L	*Aspergillus aculeatus*	3.5–5.5	25–55	Endoglucanase (100 FGU/g), pectinase, xylanases, and hemicellulase
Celluclast 1.5L	*Trichoderma reesei*	4–6	25–55	Cellulase activity (700 EGU/g), xylanase (680 U/mL) and endoglucanase (26 U/mL)
Pectinase Ultra Tropical	*Aspergillus sp.*	3–5	10–55	Pectinase (5000 PECTU/g), polygalacturonase, pectin lyase, cellulase, xylanase and endo-1,4-β-glucanase
Shearzyme Plus 2X	*Aspergillus oryzae/ Trichoderma reesei*	4–5.5	25	Cellulase (350 EGU/g), endo-1,4-b-xylanase (250 FXU/g) and a side activity of β-glucanase

BGU: fungal β-glucanase units; EGU: endoglucanase units; FXU: fungal endoxylanase units; CIU: cellulase international units; PECTU: pectinase units; FGU: Fungal glucasase units;

Table S2. Retention time (Rt), wavelengths of maximum absorption in the visible region (λ_{max}), mass spectral data and tentative identification of phenolic compounds in raw and processed lentil hulls

Peak	Rt (min)	λ_{max} (nm)	Molecular ion [M-H]⁻ *m/z*	MS² (*m/z*)	Tentative identification
1	4.20	270	169	125	Gallic acid
2	4.65	278	897	593, 289	Trimer prodelphinidin
3	4.99	278	593	425, 289	Dimer prodelphinidin (I)
4	5.30	278	881	577,525,289	Galloylated dimer (I)
5	5.53	278	451	289	(+)-Catechin *O*-hexoside
6	5.80	277	577	289	Dimer procyanidin (I)
7	5.99	278	593	425, 289	Dimer prodelphinidin (II)
8	6.20	278	593	425, 289	Dimer prodelphinidin (III)
9	6.54	278	577	425, 289	Dimer procyanidin (II)
10	6.63	278	865	577, 525, 289	Trimer procyanidin
11	7.47	278	289	245, 137	(+)-catechin
12	7.96	280	881	577,525,289	Galloylated dimer (II)
13	9.26	279	289	245, 137	(-)-epicatechin
14	10.80	306	267	163	*trans*-*p*-coumaric acid derivative (I)
15	11.45	310	267	163	*trans*-*p*-coumaric acid derivative (II)
16	12.68	347	901	755,609,285	Kaempferol dirutinoside
17	12.81	346	755	609,285	Kaempferol rutinoside hexoside
18	13.50	350	771	609,591,300	Quercetin rutinoside hexoside

Peaks 1, 11 and 13 were identified by comparison of their retention times and UV spectra with commercial standards.

Peaks 2-4, 6-10 and 12 showed a UV spectra similar to procyanidin. These compounds presented a pseudomolecular ion [M-H]⁻ at 897,593,881,577 and 865, releasing and MS2 fragment a *m/z* 289, corresponding to a (epi)-catechin monomer.

Peak 5 showed a pseudomolecular ion M-H]$^-$ at 451, releasing an MS2 fragment at *m/z* 289 (M-H-162]$^-$ loss of an hexosyl moiety), corresponding to a catechin monomer. This is compound was tentatively identified as (+)-catechin *O*-hexoside. Peak 14 and 15 presented UV spectra similar to *trans-p*-coumaric acid but with different retention times and confirmed with the fragment [M-H]$^-$ at *m/z* 163 from *p*-coumaric acid. These compounds are identified as a *trans*–*p*-coumaric acid derivatives.

Peak 16 and 17 presented a UV spectrum (λmax 346) corresponding to the flavanol kaempferol, which showed a molecular ion [M-H]- at 901 and 755, and a fragment ion [M-H]- at *m/z* 285, corresponding to kaempferol.

Peak 18 presented a UV spectrum (λmax 350) corresponding to the flavanol quercetin, which showed a molecular ion [M-H]- at 771, and a fragment ion [M-H]- at *m/z* 300 [M-308-162]$^{-,}$ loss of rutinoside and hexoside molecules), corresponding to quercetin.

All these compounds were previously identified in lentils by Aguilera et al., [1], Bautista-Exposito et al., [2] and seed coat of lentils by Dueñas et al., [3].

References

[1] Aguilera, Y.; Dueñas, M.; Estrella, I., Hernández, T., Benitez, V., Esteban, R.M.; Martín-Cabrejas, M.A. Evaluation of phenolic profile and antioxidant properties of pardina lentils as affected by industrial dehydration. *Journal of Agricultural and Food Chemistry* **2010**, 58, 10101-10108, doi: 10.1021/jf102222t
[2] Bautista-Expósito, S.; Peñas, E.; Dueñas, M.; Silván, J.M.; Frias, J.; Martínez-Villaluenga, C. Individual contributions of Savinase and Lactobacillus plantarum to lentil functionalization during alkaline pH-controlled fermentation. *Food Chemistry* **2018**, *257*, 341-349, doi:10.1016/j.foodchem.2018.03.044.
[3] Dueñas, M.; Baoshan S., Hernández, T., Estrella, I., Spranger, M.I. Proanthocyanidin composition in the seed coat of lentils (*Lens culinaris* L.). *Journal of Agricultural and Food Chemistry* **2003,** 51, 7999-8004. , doi: 10.1021/jf10303215
